# Agreement between PDL1 immunohistochemistry assays and polymerase chain reaction in non-small cell lung cancer: CLOVER comparison study

**DOI:** 10.1038/s41598-020-60950-2

**Published:** 2020-03-03

**Authors:** Ilya Tsimafeyeu, Evgeny Imyanitov, Larisa Zavalishina, Grigory Raskin, Patrisia Povilaitite, Nikita Savelov, Ekaterina Kharitonova, Alexey Rumyantsev, Inna Pugach, Yulia Andreeva, Alexey Petrov, Georgy Frank, Sergei Tjulandin

**Affiliations:** 1 Russian Society of Clinical Oncology, Moscow, Russia; 2 N.N. Petrov Institute of Oncology, St. Petersburg, Russia; 3 Russian Medical Academy of Continuous Professional Education, Moscow, Russia; 4 A.M. Granov Russian Scientific Center of Radiology and Surgical Technologies, St. Petersburg, Russia; 5 Rostov Regional Bureau of Pathology, Rostov-on-Don, Russia; 6 Moscow City Oncology Hospital №62, Moscow, Russia; 7 N.N. Blokhin Russian Cancer Research Centre, Moscow, Russia; 8 Present Address: Presented in part at the 2018 ESMO Congress, Munich, Germany

**Keywords:** Applied immunology, Immunotherapy, Tumour immunology, Cancer therapy, Lung cancer, Tumour biomarkers

## Abstract

The goal of the CLOVER study was to perform a pairwise comparison of four tests based on the same patient population with non-small cell lung cancer (NSCLC): three validated PDL1 immunohistochemistry (IHC) assays (Ventana SP142, Ventana SP263, Dako 22C3) and one PCR test. Four hundred seventy-three NSCLC samples were obtained from a biobank and were stained using PDL1 IHC assays. Four trained pathologists independently evaluated the percentage of tumor cells (TC) and immune cells (IC) that stained positive at any intensity. *PDL1* transcripts were quantified in 437 patients by a standard Taqman RT-PCR assay using *SDHA* as a reference gene. A concordance analysis was performed to assess (1) the correlation of TC and IC between different assays and (2) the predictive properties of one test for another. “High” RNA expression was detected in 187 of 437 (43%) patients. The percentage of PDL1-positive cells (≥1%) was higher among the IC than the TC in all IHC three assays. The Pearson correlation coefficients (PCC) for TC were 0.71, 0.87, and 0.75 between 22C3/SP142, 22C3/SP263, and SP263/SP142, respectively. The PCC for IC were 0.45, 0.61, and 0.68 for the same pairs. A low correlation was observed between the PCR test and each of the three IHC assays; however, if a patient tested low/negative by PCR, then they were likely to test negative by any single IHC test with a high probability (92–99%). Among patients who tested positive by PCR, only 9–45% tested positive by IHC assays. There was excellent positive and negative agreement (>91%) between 22C3 and SP263 staining using the recommended individual cutoffs for first-line treatment. PCR RNA expression analysis is not equivalent to IHC. However, this method may have some potential for the identification of PDL1-negative tumors. 22C3 could be considered as a substitute for SP263 in first-line treatment.

## Introduction

In recent years, immune checkpoint inhibitors targeting programmed cell death 1 (PD-1) or programmed cell death ligand 1 (PDL1, CD274), have presented an alternative revolutionary therapeutic approach for patients with nonsquamous and squamous non-small-cell lung cancer (NSCLC)^1^.

Pembrolizumab, an anti-PD-1 humanized antibody, is recommended as a first-line single agent for patients with metastatic NSCLC and PDL1 expression levels greater than 50% detected by immunohistochemistry (IHC) with the diagnostic antibody 22C3 (Agilent)^2,3^. Pembrolizumab was recently approved for use in the first-line setting for metastatic nonsquamous NSCLC in combination with pemetrexed and carboplatin independent of PDL1 expression^4^, or as a second-line single agent for patients with PDL1 expression levels of 1% or more^5^.

Atezolizumab, an anti-PDL1 humanized antibody, is approved in combination with chemotherapy and bevacizumab as a first-line treatment for patients with nonsquamous metastatic NSCLC regardless of PDL1 expression levels^6^. A National Comprehensive Cancer Network panel has recommended atezolizumab as subsequent therapy for patients with nonsquamous and squamous advanced NSCLC based on the results of a phase 3 trial and mentions that PDL1 testing is not required but may provide useful information^7^. In this OAK study, patients with high PDL1 expression derived the greatest benefit from atezolizumab compared with docetaxel^8^. A high expression level was defined as PDL1 expression in 50% or more of tumor cells (TC) and in 10% or more of tumor-infiltrating immune cells (IC) using diagnostic IHC antibody SP142 (Ventana Medical Systems).

Nivolumab, an anti-PD-1 human antibody, showed activity when used as subsequent therapy in all patient population with nonsquamous and squamous NSCLC regardless of the level of PDL1 expression^9^. Finally, durvalumab, an anti-PDL1 human antibody, is approved as consolidation therapy for patients with unresectable stage III NSCLC who have not progressed after 2 or more cycles of definitive concurrent platinum-based chemoradiation^10^. The progression-free survival benefit with durvalumab was observed irrespective of PDL1 status (<25% or ≥25% of expressed tumor cells stained by diagnostic antibody SP263 (Ventana Medical Systems) before chemoradiotherapy.

While the IHC assessment of PDL1 expression in tumor samples has emerged as a possible biomarker of susceptibility to immune checkpoint inhibitors, its potential use poses many questions and challenges for both oncologists and pathologists. Different methods of interpretation and cutoff values are used for each antibody to determine the PDL1 status of a tumor and to predict its response to immunotherapy in clinical studies and routine practice. Many pathology departments do not currently have all of the automated platforms and reagents for the various *in vitro* diagnostic assays. Furthermore, detection of PDL1 expression is required after molecular testing (EGFR, ALK) performed by polymerase-chain reaction (PCR) that could affect the duration of molecular diagnosis and its cost. There have been no large studies that investigated the assessment of PDL1 expression levels by PCR and compared this with IHC assays.

The goal of this study conducted by the Russian Society of Clinical Oncology (RUSSCO) was to perform a pairwise comparison of four tests based on the same patient population: one PCR test and three validated PDL1 IHC assays (22C3, SP142, and SP263; CLOVER study).

## Methods

### Tumor samples

For this study, 500 archived NSCLC samples (formalin-fixed, paraffin-embedded blocks) were provided by the RUSSCO biobank. Informed consent was obtained from all subjects. Four hundred seventy-three specimens contained sufficient material for expression assays (27 samples were excluded from the final analysis as they did not meet sample requirements for IHC, and 36 samples were excluded from the PCR testing). The sample age ranged from 0.5 to 1 year, based on the date of excision. These samples were not associated with any clinical studies or immune checkpoint inhibitor therapy.

Consecutive sections were used to reduce the variability between assays due to tumor heterogeneity. Four sections of each tumor specimen were prepared for examination; additional sections were prepared for use as negative controls. We used the cell line NCL-H226 as a positive control, the cell line MCF-7 as a negative control, as well as tonsillar tissue (for 22С3) and placental tissue (for SP142 and SP263) samples as positive controls in each assay cycle^11^. First, we verified assays using the positive and negative cell lines as well as positive tissue controls. Then, we performed IHC staining on the NSCLC samples.

Slides (N = 1,419) were stained with the anti-PDL1 IHC antibodies as was done in the clinical trials of therapy with pembrolizumab (clone 22C3; Agilent), atezolizumab (clone SP142; Ventana Medical Systems), and durvalumab (clone SP263; Ventana Medical Systems)^3,8,10^. The antibodies were used in automated IHC assays. Clone 22С3 was tested with the Dako Autostainer Link 48 (Agilent) using the optimized closed protocol provided by the manufacturer for the automated platform. Assays with SP142 were performed with the BenchMark ULTRA staining instrument (Ventana Medical Systems), according to the protocols included in the instructions for use of the antibodies, and the external quality control system from Nordic immunohistochemical Quality Control (NordiQC) for SP263. We detected antibody staining with the OptiView DAB IHC Detection Kit with (for the SP142 antibody) and without (for the SP263 antibody) the OptiView Amplification Kit (Ventana Medical Systems) in accordance with the protocols recommended by the manufacturer. Four trained pathologists, certified by Ventana/Roche and Dako/Agilent for the interpretation of the respective assays, independently evaluated the percentages of TC and IC that stained positive at any intensity for PDL1 expression. When the interpretations differed, the pathologists made consensus decisions. According to the PDL1 expression assessment recommendations, positive membrane staining, irrespective of its intensity, was evaluated in TC and IC.

PDL1 transcripts were quantified in 437 of those patients using a standard Taqman reverse transcription PCR (RT-PCR) assay with *SDHA* as the reference gene.

All methods were carried out in accordance with relevant guidelines and regulations. The study protocol was approved by the principal investigators and Russian Society of Clinical Oncology (RUSSCO) independent ethics committee (approval number 08122016). All patients provided their written informed consent.

### Data analysis

We assessed the correlations between the results from the different assays determining PDL1 expression in TC or IC. Four pairwise comparisons of the relative frequencies of two tests indicating positive or negative staining/RNA expression were conducted to estimate the probability of agreement or disagreement between each pair of tests. We estimated the conditional probability of one test indicating positive or negative staining/RNA expression given the outcome of each other test.

One test-specific cutoff rule for each assay was pre-specified as: for first-line treatment the Tumor Proportion Score (TPS, the percentage of viable tumor cells showing partial or complete membrane staining relative to all viable tumor cells present in the sample) ≥50% for 22C3, TC or IC ≥ 5% for SP142, TC ≥ 25% for SP263, and for second-line treatment TPS ≥ 1% for 22C3, TC ≥ 50% or IC ≥ 10% for SP142, and TC ≥ 25% for SP263 (Table 1). Delta CT = 2 was conditionally chosen as the threshold between “high” (“positive”) and “low/absent” (“negative”) *PDL1* RNA expression.

**Table 1:** Test-specific cutoff rule for each IHC assay (PDL1 positivity).

Treatment line	22C3	SP142		SP263
	TPS	TC or IC		TC
First-line	≥50%	≥5%	≥5%	≥25%
Second-line	≥1%	≥50%	≥10%	≥25%

TPS - Tumor Proportion Score, TC - tumor cells, IC - immune cells.

## Results

### Patient characteristics

The patients were predominantly male (68%) with a mean age of 61.3 years (range: 28–85 years). Samples were from patients with stage I–IV NSCLC. Tumor stages (T) 1, 2, 3, and 4 were observed in 78 (16.5%), 137 (29%), 63 (13.3%), and 195 (41.2%) patients, respectively. Lymph node metastases were found in 353 (75%) patients; 236 (50%) patients presented with distant metastases. NSCLC samples included 81 (17%) EGFR-positive, 37 (8%) ALK-positive, and 91 (19%) squamous-cell carcinoma specimens. None of the patients received radio- or systemic therapy before surgical excision.

### PDL1 expression

“High” *PDL1* RNA expression was detected in 187 of 437 (43%) patients.

PDL1 staining without using any cutoff was observed in both TC and IC by all three of the PDL1IHC assays. The percentages of PDL1-positive cells (≥1%) were higher for the IC than the TC in all three assays (54% versus 39% for 22C3, 49% versus 21% for SP142, and 69% versus 51% for SP263). The staining results varied for the IC and TC between the SP142 and the other assays.

For each of the assays, we also performed these analyses at the IHC cutoffs of ≥10%, ≥25%, and ≥50% PDL1-positive cells among the TC and IC. With a cutoff for positive staining of ≥10% of TC, the highest proportion of positive samples was observed after staining with the 22С3 (21%) and SP263 (20%) antibodies, whereas staining with the SP142 antibody indicated that only 8% of samples contained TC were PDL1-positive. For the higher cutoffs (≥25%, and ≥50%), similar results were obtained. With a cutoff for positive staining of ≥25% of TC, the 22C3 and SP263 antibodies indicated that 16% and 15% of samples had PDL1-positive TC, respectively. The SP142 antibody stained only 5% of samples. Staining with 22С3 and SP263 detected ≥50% of PDL1-positive TC in 12% and 10% of patients, respectively, whereas staining with SP142 detected 3% of PDL1-positive TC.

**Figure 1:**
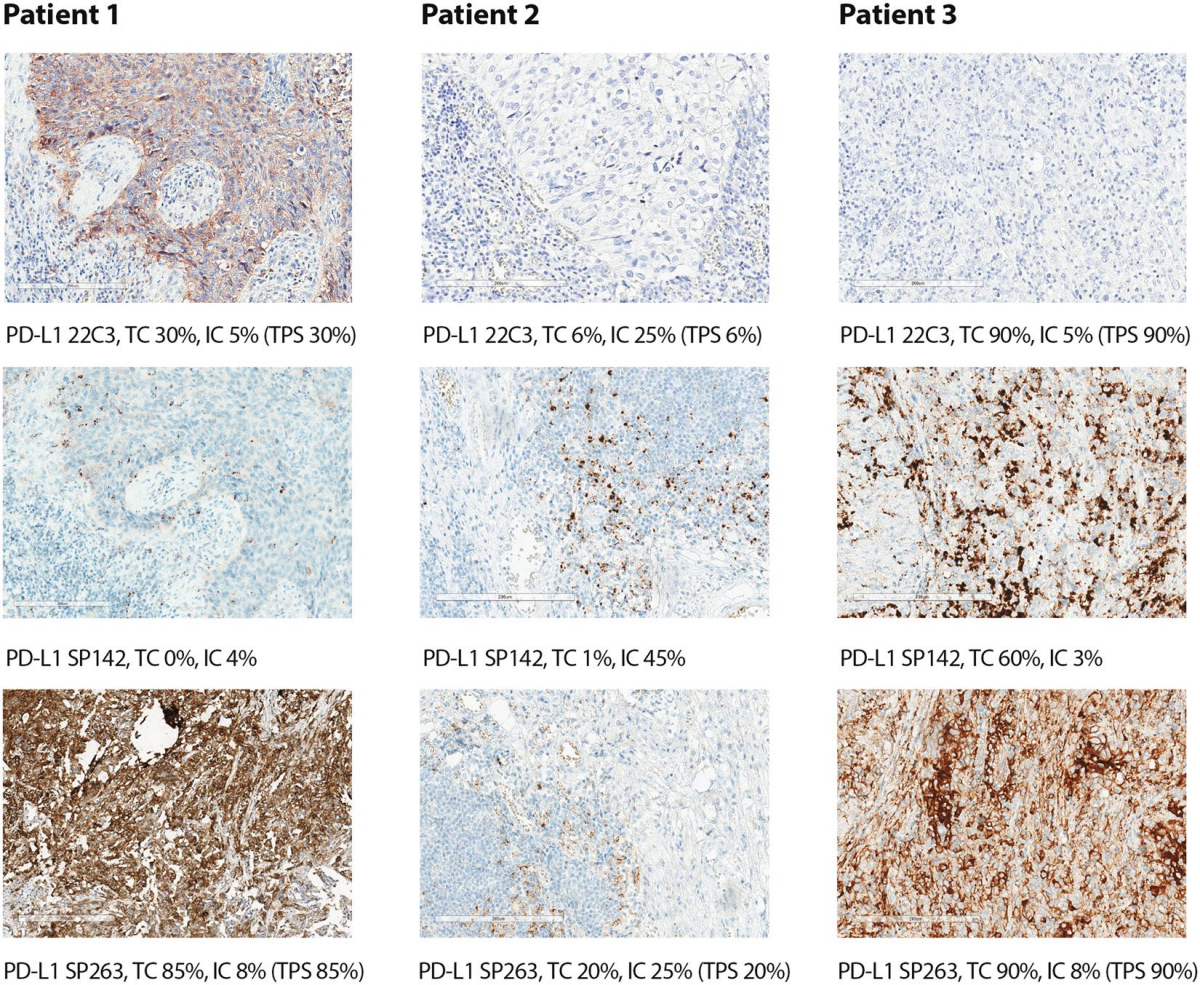
Representative images of sample stained with the three IHC diagnostic assays.

The highest proportion of samples with PDL1-positive IC using the ≥10% cutoff was observed after staining with the 22C3 (11%) and SP263 (9%) antibodies. The SP142 antibody indicated that 4% of samples contained IC that were PDL1-positive. The use of the higher cutoffs dramatically decreased the proportion of samples classified as containing PDL1-positive IC; with a cutoff of ≥25%, only 1.1% (22C3), 0.6% (SP142), and 0.8% (SP263) of cases were considered positive. Further increases in the cutoff value resulted in even lower proportions of samples categorized as containing PDL1-positive IC (0–0.2%). Figure 1 provides representative images of the IHC staining with the three antibodies.

**Figure 2:**
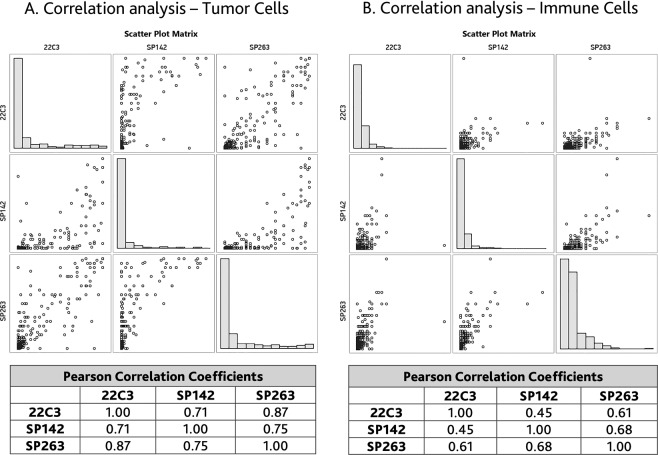
Correlations of the percentages of TC and IC PDL1 membrane staining in NSCLC samples.

**Figure 3:**
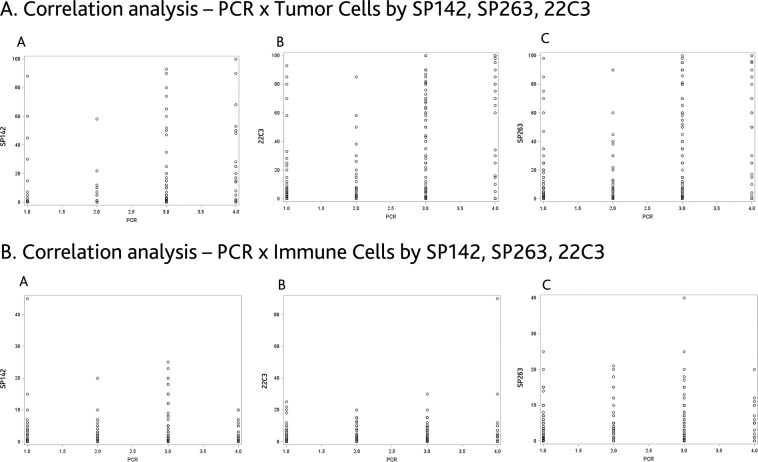
Correlations of the percentages of TC and IC PDL1 membrane staining and PCR expression in NSCLC samples.

Further analysis was performed to assess the correlation of TC and IC between different IHC assays and PCR (Figs. 2,3). The Pearson correlation coefficients (PCC) for TC were 0.71, 0.87 and 0.75 between 22C3/SP142, 22C3/SP263, and SP263/SP142, respectively. The PCC for IC were 0.45, 0.61, and 0.68 for the same pairs. A low correlation was observed between the PCR test and any of the IHC assays for TC and IC. For example, the PCC between PCR and 22C3 for TC and IC were 0.36 and 0.14, respectively.

Table 2 presents information on PDL1 expression in patients with different EGFR and ALK status, as well as histology. Thirty-eight of the 79 (48%) patients with EGFR mutations expressed intratumoral *PDL1* RNA. Of the 81 EGFR-positive tumors, immunohistochemical PDL1 expression (≥1%) ranged from 24% to 56% for TC and from 48% to 74% for IC. With a positive staining cutoff of ≥1% for TC, 46% of ALK-positive patients were PDL1-positive when evaluated with 22C3 or SP263 antibodies as well as with a PCR test. Of the 91patients with squamous-cell carcinoma, PDL1 expression was detected in 20–76% of cases. The 22C3 and SP263 antibodies showed higher expression on TC and IC compared with the other two assays.

**Table 2:** PDL1 expression in patients with different EGFR status, ALK status, and histology.

	PCR expression	22C3		SP142		SP263	
		TC ≥ 1%	IC ≥ 1%	TC ≥ 1%	IC ≥ 1%	TC ≥ 1%	IC ≥ 1%
**EGFR-positive**	38/79 (48%)	31/81 (38%)	39/81 (48%)	19/81 (23%)	47/81 (58%)	45/81 (56%)	60/81 (74%)
**EGFR-negative**	149/358 (42%)	153/392 (39%)	217/392 (55%)	82/392 (21%)	187/392 (48%)	196/392 (50%)	264/392 (67%)
**ALK-positive**	17/37 (46%)	17/37 (46%)	12/37 (32%)	13/37 (35%)	14/37 (38%)	17/37 (46%)	12/37 (32%)
**ALK-negative**	105/298 (35%)	106/335 (32%)	154/335 (46%)	51/335 (15%)	133/335 (40%)	105/335 (31%)	154/335 (46%)
**Squamous cell carcinoma**	23/86 (27%)	42/91 (46%)	48/91 (53%)	18/91 (20%)	30/91 (33%)	57/91 (63%)	69/91 (76%)

PCR - polymerase chain reaction, TC - tumor cells, IC - immune cells, EGFR - epidermal growth factor receptor mutations, ALK - anaplastic lymphoma kinase rearrangements.

The proportions of PD-L1-positive cases by any of the three IHC assays or PCR test using corresponding recommended cutoff were non-significant higher among patients with stage T ≥ 3 disease compared with the proportion among patients with stage T ≤ 2 disease (all P > 0.05).

### Agreement between PDL1 diagnostic assays

We calculated the percentage agreement between results based on the pre-specified, clinically relevant expression cutoff. If a tumor had “low/absent” *PDL1* expression by PCR, then there was a high probability (91–99%) that this case was also negative by any single IHC test using the corresponding recommended cutoff. However, among the patients who were positive by PCR, only 9–45% of them were positive by IHC assays (Table 3). Differences were consistent between treatment lines. Table 4 represents how well one IHC assay could predict the same outcome (positivity or negativity) of another IHC assay using the recommended individual cutoff for each test. A high degree of agreement was observed between the 22C3 and SP263 assays at the cutoff described for first-line treatment, with a percentage agreement of > 91% observed for both positive and negative results.

**Table 3:** Negative and positive percentage agreement between PCR and IHC assays at clinically relevant PDL1 expression cutoff levels.

	SP142		SP263		22C3	
	First-line	Second-line	First-line	Second-line	First-line	Second-line
PCR	**Probability of negative IHC assay, given negative PCR**					
	91%	97%	91%	91%	97%	70%
	**Probability of positive IHC assay, given positive PCR**					
	27%	9%	26%	26%	17%	45%

PCR - polymerase chain reaction, IHC – immunohistochemistry.

**Table 4:** Negative and positive percentage agreement between IHC assays at clinically relevant PDL1 expression cutoff levels.

Probability of negative Test B, given negative Test A						
Test A	Test B					
	SP142		SP263		22C3	
	First-line	Second-line	First-line	Second-line	First-line	Second-line
SP142	—	—	92%	85%	97%	65%
SP263	91%	98%	—	—	99%	76%
22C3	88%	99%	91%	99%	—	—
**Probability of positive Test B, given positive Test A**						
SP142	—	—	65%	76%	48%	94%
SP263	68%	28%	—	—	57%	98%
22C3	82%	17%	93%	49%	—	—

PCR - polymerase chain reaction, IHC – immunohistochemistry.

### Independent pathology review

The percentage agreement at the clinically relevant expression cutoff between the original laboratory pathology results and the independent pathologist review was >87% for all assays.

## Discussion

This was the largest harmonization study performed to date to establish the extent of analytic concordance between the PCR test and three validated PDL1 IHC diagnostic assays that have been used in randomized clinical trials of checkpoint inhibitors in patients with NSCLC. Evaluation of PDL1 expression immediately after the assessment of EGFR mutations and ALK/ROS1 rearrangements in a single molecular biology laboratory could reduce the duration and cost of testing^12^. Interchangeable IHC assays could also facilitate the assessment of protein expression in routine clinical practice^11^.

*PDL1* RNA expression was detected by PCR in a large number of patients (>40%), which may seem comparable with the evaluation by IHC. Similar PCR and IHC results for expression frequency were obtained in an analysis of patients with EGFR-positive and EGFR-negative lung cancer. In our study, the frequency of *PDL1* expression determined by PCR is consistent with the data obtained in the smaller Isobe study^13^. In addition, we showed that among patients who were negative by PCR, more than 92% were negative by each of the three IHC assays using the corresponding recommended cutoff. However, PCR had a high probability of false-positive prediction and very low correlation coefficients for both TC and IC. Thus, the results of our study suggest that significant differences exist between the PCR test and three PDL1 IHC assays and, as such, they are not comparable enough to use interchangeably.

When comparing the results of IHC assays, the most consistent results were obtained for the pairwise comparison of 22C3 and SP263. In agreement with previous studies^14–17^, SP142 showed consistently lower scores than the other two assays. Therefore, 22C3 could be considered as a substitute for SP263 in first-line treatment. There was excellent correlation between the 22C3 and SP263 staining, with a high PCC of 0.87 for the TC score. The results from the Blueprint PDL1 IHC Comparability Project demonstrated that 22C3 and SP263 assays yielded comparable analytical performance for assessment of PDL1 expression on TC according to the correlation coefficients^14^. In our study, the positive percentage agreement among the two assays was 93%, and the negative percentage agreement was 91%. Using the same recommended expression cutoffs (SP263 ≥ 2 5%, 22C3 ≥ 50%) in a large harmonization study, Ratcliffe *et al*. found high analytical concordance (percentage positive and negative agreement, 91.7–94.1%) between 22C3 and SP263^15^. Additionally, Ratcliffe’s study showed that SP263 could be considered as a substitute for 22C3 (percentage positive and negative agreement, 86–98.8%), expanding indications for the SP263 assay to the identification of patients eligible for treatment with pembrolizumab in the first-line setting. We found only 57% of positive 22C3 in cases with positive SP263. Therefore, we should be very careful when considering possible replacement of 22С3 with SP263 in all cases. However, we can assume that if a patient is negative by SP263 or SP142 in the first-line treatment setting, retesting may not be necessary by other tests based on the high agreement (ranging from 91–97%) in our study. Generally, the positive percentage agreement among the three IHC assays (ranging from 17–98%) varied more than the negative percentage agreement (ranging from 85–99%), according to the expression cutoff used for the comparative assays. Hendry *et al*. obtained similar results^16^. All three assays (22C3, SP142, and SP263) yielded consistent results for negative agreement, but positive agreement was poor.

Regarding the cutoffs in second-line treatment, we believe that in the case of positive SP142 or SP263 assays, retesting by 22C3 may not be required. Almost all cases that scored as positive on the Ventana platform (94–98%) also scored as positive on the Dako platform. For the same reason, retest in initially 22C3-negative samples by SP142 or SP263 may also not be necessary (pairwise agreement between all pairs of tests was 99%).

PDL1 expression in TC in patients with EGFR-negative and EGFR-positive NSCLC was very similar regardless of the evaluation method. In contrast, more significant differences in PDL1 expression were found in IC. Regarding ALK translocation, which is determined in 8% of Russian patients^18^, the numbers in each category are rather small for interpretation. However, patients with ALK-positive lung cancer were more likely to express PDL1 in TC than patients without ALK rearrangements. PCR and SP142 showed weaker expression in TC and IC compared with the other two assays in patients with squamous cell carcinoma. The agreement analysis for the PDL1-positive tumor status did not reveal any marked differences between the clones and PCR.

In conclusion, results of the CLOVER study demonstrate a low agreement between PDL1 PCR and IHC expression in NSCLC. PCR RNA expression analysis is not equivalent to IHC methods; however, it may have some potential for identifying PDL1-negative tumors. We suggest that the 22C3 assay could predict the same outcome (positivity or negativity) of the SP263 assay in patients before first-line therapy with a checkpoint inhibitor. Patients classified as negative bySP263 or SP142 assays using the corresponding cutoff rule for first-line treatment are highly likely to be classified as negative by any other test. In patients with positive SP263 and SP142 or negative 22C3 repeated testing in second-line treatment could be avoided. The current assessment criteria may change with regard to different cutoff values established from the emerging results of new clinical studies.

## References

[1] Rangachari Deepa, Costa Daniel B. (2019). From Hope to Reality: Durable Overall Survival With Immune Checkpoint Inhibitors for Advanced Lung Cancer. Journal of Clinical Oncology.

[2] Garon Edward B., Hellmann Matthew D., Rizvi Naiyer A., Carcereny Enric, Leighl Natasha B., Ahn Myung-Ju, Eder Joseph Paul, Balmanoukian Ani S., Aggarwal Charu, Horn Leora, Patnaik Amita, Gubens Matthew, Ramalingam Suresh S., Felip Enriqueta, Goldman Jonathan W., Scalzo Cathie, Jensen Erin, Kush Debra A., Hui Rina (2019). Five-Year Overall Survival for Patients With Advanced Non‒Small-Cell Lung Cancer Treated With Pembrolizumab: Results From the Phase I KEYNOTE-001 Study. Journal of Clinical Oncology.

[3] Mok, T. S. K. *et al*. Pembrolizumab versus chemotherapy for previously untreated, PD-L1-expressing, locally advanced or metastatic non-small-cell lung cancer (KEYNOTE-042): a randomised, open-label, controlled, phase 3 trial. *Lancet*. May 4, **393**(10183), 1819–1830 (2019). 10.1016/S0140-6736(18)32409-7 30955977

[4] U.S. Food & Drug Administration. FDA approves pembrolizumab in combination with chemotherapy for first-line treatment of metastatic squamous NSCLC, https://www.fda.gov/drugs/fda-approves-pembrolizumab-combination-chemotherapy-first-line-treatment-metastatic-squamous-nsclc Accessed August 12, (2019).

[5] Pai-Scherf L (2017). FDA Approval Summary: Pembrolizumab for Treatment of Metastatic Non-Small Cell Lung Cancer: First-Line Therapy and Beyond. Oncologist..

[6] Laktionov K. K., Sarantseva K. A., Breder V. V., Okruzhnova M. A. & Peregudova M. V. Immunotherapy for non-small cell lung cancer treatment. Malignant tumours; **3**,17–24. (In Russ.). (2016).

[7] NCCN Clinical Practice Guidelines in Oncology. Non-small cell lung cancer. Version 5, https://www.nccn.org/professionals/physician_gls/PDF/nscl.pdf. (2019)

[8] Rittmeyer, A. *et al*. Atezolizumab versus docetaxel in patients with previously treated non-small-cell lung cancer (OAK): a phase 3, open-label, multicentre randomised controlled trial. *Lancet*. Jan 21, **389**(10066), 255–265 (2017). 10.1016/S0140-6736(16)32517-X PMC6886121 27979383

[9] Kazandjian Dickran, Suzman Daniel L., Blumenthal Gideon, Mushti Sirisha, He Kun, Libeg Meredith, Keegan Patricia, Pazdur Richard (2016). FDA Approval Summary: Nivolumab for the Treatment of Metastatic Non‐Small Cell Lung Cancer With Progression On or After Platinum‐Based Chemotherapy. The Oncologist.

[10] Antonia, S. J. *et al*. Durvalumab after Chemoradiotherapy in Stage III Non-Small-Cell Lung Cancer. *N. Engl. J. Med*. Nov. 16; **377**(20), 1919–1929 (2017). 10.1056/NEJMoa1709937 28885881

[11] Zavalishina Larisa, Tsimafeyeu Ilya, Povilaitite Patrisia, Raskin Grigory, Andreeva Yulia, Petrov Alexey, Kharitonova Ekaterina, Rumyantsev Alexey, Pugach Inna, Frank Georgy, Tjulandin Sergei (2018). RUSSCO-RSP comparative study of immunohistochemistry diagnostic assays for PD-L1 expression in urothelial bladder cancer. Virchows Archiv.

[12] Imyanitov, E. N. *et al*. Distribution of EGFR Mutations in 10,607 Russian Patients with Lung Cancer. *Mol. Diagn. Ther*. Aug; **20**(4), 401–6 (2016). 10.1007/s40291-016-0213-4 27259329

[13] Isobe, K. *et al*. PD-L1 mRNA expression in EGFR-mutant lung adenocarcinoma. *Oncol. Rep*. Jul, **40**(1), 331–338 (2018). 10.3892/or.2018.6442 29767258

[14] Tsao, M. S. *et al*. PD-L1 Immunohistochemistry Comparability Study in Real-Life Clinical Samples: Results of Blueprint Phase 2 Project. *J. Thorac. Oncol*. Sep, **13**(9), 1302–1311 (2018). 10.1016/j.jtho.2018.05.013 PMC8386299 29800747

[15] Ratcliffe Marianne J., Sharpe Alan, Midha Anita, Barker Craig, Scott Marietta, Scorer Paul, Al-Masri Hytham, Rebelatto Marlon C., Walker Jill (2017). Agreement between Programmed Cell Death Ligand-1 Diagnostic Assays across Multiple Protein Expression Cutoffs in Non–Small Cell Lung Cancer. Clinical Cancer Research.

[16] Hendry Shona, Byrne David J., Wright Gavin M., Young Richard J., Sturrock Sue, Cooper Wendy A., Fox Stephen B. (2018). Comparison of Four PD-L1 Immunohistochemical Assays in Lung Cancer. Journal of Thoracic Oncology.

[17] Rimm DL (2017). A prospective, multiinstitutional, pathologist-based assessment of 4 immunohistochemistry assays for PD-L1 expression in non-small cell lung cancer. JAMA Oncol..

[18] Tsimafeyeu, I. *et al*. Overall Survival of Patients With ALK-Positive Metastatic Non-Small-Cell Lung Cancer in the Russian Federation: Nationwide Cohort Study. *J. Glob. Oncol*. May, **5**, 1–7 (2019). 10.1200/JGO.19.00024 PMC6550093 31095455

